# Research Progress on Cloning and Function of *Xa* Genes Against Rice Bacterial Blight

**DOI:** 10.3389/fpls.2022.847199

**Published:** 2022-03-21

**Authors:** Yong Yang, Yuhang Zhou, Jia Sun, Weifang Liang, Xinyu Chen, Xuming Wang, Jie Zhou, Chulang Yu, Junmin Wang, Shilu Wu, Xiaoming Yao, Yujie Zhou, Jie Zhu, Chengqi Yan, Bingsong Zheng, Jianping Chen

**Affiliations:** ^1^State Key Laboratory for Managing Biotic and Chemical Treats to the Quality and Safety of Agro-Products, Key Laboratory of Biotechnology for Plant Protection, Ministry of Agriculture, and Rural Affairs, Zhejiang Provincial Key Laboratory of Biotechnology for Plant Protection, Institute of Virology and Biotechnology, Zhejiang Academy of Agricultural Science, Hangzhou, China; ^2^State Key Laboratory of Subtropical Silviculture, Zhejiang A & F University, Hangzhou, China; ^3^College of Plant Protection, Fujian A & F University, Fuzhou, China; ^4^College of Plant Protection, Yunnan Agricultural University, Kunming, China; ^5^State Key Laboratory for Managing Biotic and Chemical Treats to the Quality and Safety of Agro-Products, Key Laboratory of Biotechnology for Plant Protection, Ministry of Agriculture, and Rural Affairs, Zhejiang Provincial Key Laboratory of Biotechnology for Plant Protection, Institute of Plant Virology, Ningbo University, Ningbo, China; ^6^College of Agronomy, Anhui Agricultural University, Hefei, China; ^7^Zhejiang Plant Protection, Quarantine and Pesticide Management Station, Hangzhou, China; ^8^Zhuji Agricultural Technology Extension Center, Zhuji, China; ^9^Plant Protection and Soil Fertilizer Management Station of Wenzhou, Wenzhou, China; ^10^Institute of Biotechnology, Ningbo Academy of Agricultural Science, Ningbo, China

**Keywords:** rice bacterial blight, resistance genes, gene mapping, map-based cloning, resistance mechanism, resistance breeding

## Abstract

Bacterial blight (BB) of rice caused by *Xanthomonas oryzae* pv. *oryzae* (*Xoo*) is one of the most serious bacterial diseases that hinder the normal growth and production of rice, which greatly reduces the quality and yield of rice. The effect of traditional methods such as chemical control is often not ideal. A series of production practices have shown that among the numerous methods for BB controlling, breeding and using resistant varieties are the most economical, effective, and environmentally friendly, and the important basis for BB resistance breeding is the exploration of resistance genes and their functional research. So far, 44 rice BB resistance genes have been identified and confirmed by international registration or reported in journals, of which 15 have been successfully cloned and characterized. In this paper, research progress in recent years is reviewed mainly on the identification, map-based cloning, molecular resistance mechanism, and application in rice breeding of these BB resistance genes, and the future influence and direction of the remained research for rice BB resistance breeding are also prospected.

## Introduction

As a food crop closely related to human beings, rice is related to the food needs of many people in China and the world. Relevant data show that the annual yield of rice accounts for about 43% of China’s total grain output, and it is an important food crop in China. Therefore, stable yield and yield increase in rice are important measures to ensure food security. However, in the growth process of rice, many diseases inevitably occur. On the one hand, the occurrence of these diseases seriously reduces the yield and quality of rice, and on the other hand, it also restricts the development of social economy.

Bacterial blight (BB) of rice caused by Gram-negative bacteria *Xanthomonas oryzae* pv. *oryzae* (*Xoo*) is one of the most serious bacterial diseases that hinder the normal growth and production of rice and is a global bacterial disease of rice (Gnanamanickam et al., 1999). Rice BB is a kind of bacterial disease. Although it is different from fungal spores or rice planthoppers and other pests by means of atmospheric circulation remote transmission, it can be transmitted to other countries with wind, rain, or diseased rice varieties. Since its first discovery in Japan in 1884, the disease has spread to major rice-producing countries such as East Asia and Southeast Asia and has now occurred to varying degrees in Africa, the Americas, South Europe, and Australia in addition to in Asia (Gonzalez et al., 2007). Related studies have shown that the yield reduction rate of 20–30% by moderate infection of BB in the field can be as high as 50% in severe cases, or even absolute yield.

Rice BB is a typical disease in line with the “gene-to-gene” hypothesis. Due to the continuous differentiation of *Xoo* physiological races and the emergence of new virulent pathogen populations, some rice germplasm resources with resistance genes will lose their resistance after planting for a period of time (Waikhom et al., 2015). In the past century, scientists from all over the world have done a lot of research on the disease control of rice BB. However, both biological control and chemical control have failed to achieve the ideal control effect. A series of practical breeding practices have also shown that among the numerous means of BB control, the research and cultivation of resistant rice varieties are the most economical, effective, and environmentally friendly, and the foundation and key to the success of disease resistance breeding are the excavation of disease resistance genes.

## Identification and Mapping of Rice BB Resistance Genes

It is a long process from identification to mapping of rice resistance genes. As the smallest species in Gramineae, the whole genome sequencing of rice has been completed, which provides great convenience for the mapping of BB resistance genes (Yu et al., 2002).

Up to now, there are 44 rice BB resistance genes confirmed by international registration and reported in journals. Among them, 28 were dominant disease resistance genes (*Xa2* and *Xa31* belonged to the same, and *Xa3* and *Xa26* belonged to the same), 16 were recessive disease resistance genes [*xa5*, *xa8*, *xa13*, *xa15*, *xa19*, *xa20*, *xa24*, *xa25*, *xa28*, *xa32*, *xa33*, *xa34*, *xa41*, *xa42*, *xa42* (2), and *xa44*], 37 had been mapped, and 15 had been cloned (*Xa1*, *Xa2*/*Xa31*, *Xa3*/*Xa26*, *Xa4*, *xa5*, *Xa7*, *Xa10*, *xa13*, *Xa14*, *Xa21*, *Xa23*, *xa25*, *Xa27*, *xa41*, and *Xa45*).

**Figure 1:**
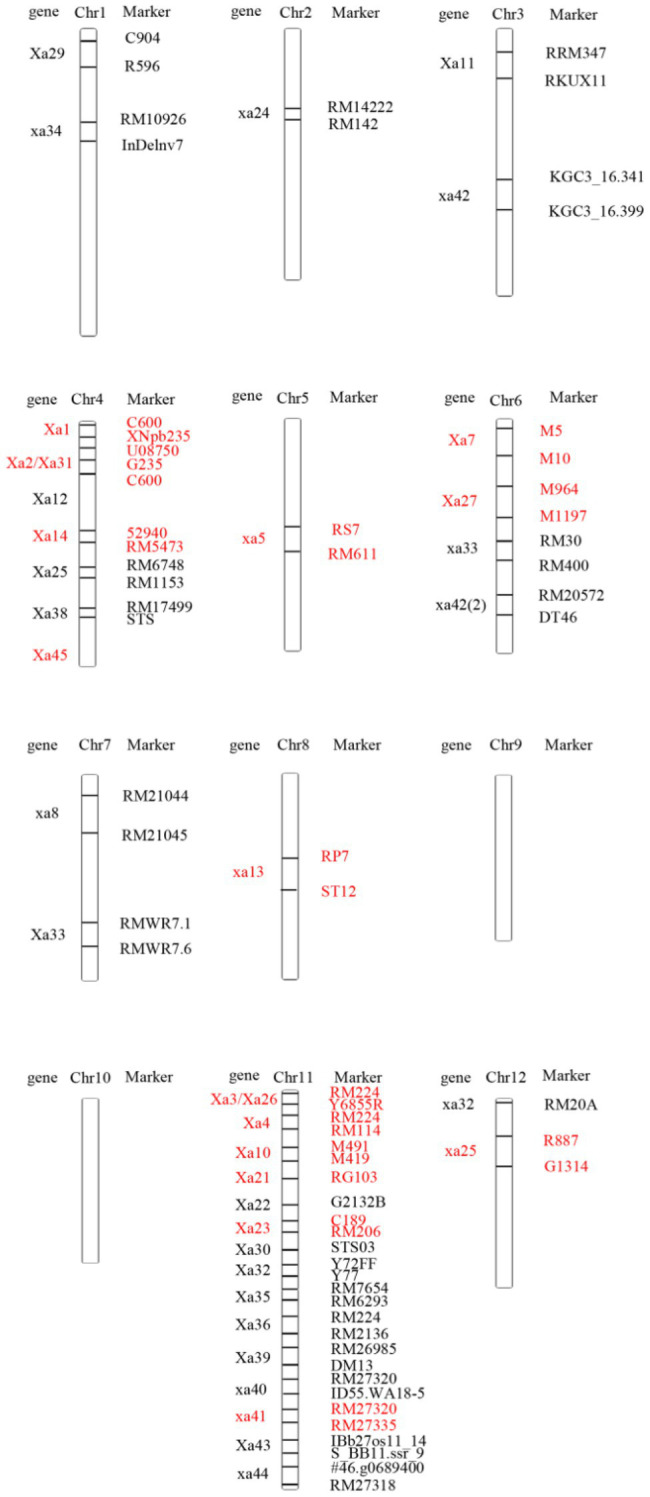
Distribution of resistance genes on rice chromosomes.

According to the location of the genes that have been mapped at present, there are different numbers of BB resistance genes on 10 chromosomes except rice chromosomes 9 and 10 (Figure 1). Most resistance genes were distributed on chromosome 11, nearly 1/3. In addition, more resistance genes were distributed on chromosomes 4 and 6. This indicated that rice BB resistance genes were clustered on chromosome. The location of these resistance genes on chromosomes laid a foundation for the deep cloning of genes and their application in rice disease resistance breeding.

### Unmapped Rice Bacterial Blight Resistance Genes

Up to now, the resistance genes to BB in rice that have not been mapped are derived from mutation and local varieties, with a total of seven genes, namely *xa15*, *Xa16*, *Xa17*, *Xa18*, *xa19*, *xa20*, and *xa28*. *xa15*, a recessive gene resistant to BB of rice, is derived from the mutant of variety M41 and has resistance to Japanese races I, II, III, and IV (Nakai et al., 2008). *Xa16*, a dominant gene resistant to BB of rice, is derived from the variety Tetep and has resistance to Japanese race V (Ogawa, 1993). *Xa17*, a dominant gene resistant to BB of rice, is derived from the variety Asominori and has resistance to Japanese race II (Ogawa, 1993). *Xa18*, a dominant gene resistant to BB of rice, was derived from Toyonishiki, Milyang 23, IR24, and was resistant to Myanmar strain H8584 (Ogawa, 1993). *xa19*, a recessive gene resistant to BB of rice, is derived from the mutant XM5 of variety IR24 and has resistance to six Filipino races (Satoru et al., 2008). *xa20*, a recessive gene resistant to BB of rice, is derived from the mutant XM6 of variety IR24 and has resistance to six Filipino races (Taura et al., 1992b). *xa28*, a recessive gene resistant to BB of rice, is derived from the variety Lota Sail and resistant to race 2 of the Philippines (Lee et al., 2003).

### Mapped Rice Bacterial Blight Resistance Genes

#### Rice Chromosome 1

Two resistance genes were located on rice chromosome 1: *Xa29* and *xa34*.

*Xa29*, a dominant gene resistant to BB of rice, was derived from the penetration line B5 of medicinal wild rice and was resistant to the Philippine race PXO61. The gene was located between RFLP markers C904 and R596 on chromosome 1, and the genetic distance was 1.3 cM (Tan et al., 2004).

*xa34*, a recessive gene resistant to rice BB, derived from Sri Lanka variety BG1222, is resistant to Chinese race 5226. The gene was located between the markers RM10929 and BGID25 on chromosome 1, with a genetic distance of 0.2 cM and a physical distance of about 204 kb, and was co-segregated with markers BGID34 and BGID36 (Shen et al., 2011). The F2 genetic segregation population was constructed using *xa34* vector variety BG1222 as the resistance donor and JG30 as the susceptible variety. The disease resistance was identified by screening the recombinants and developing the recombinant F3 family, which was finely located on chromosome 1 of rice and located in the 56.47 kb region between the molecular markers RM10926 and InDelnv7 (Shen et al., 2011).

#### Rice Chromosome 2

Only one resistance gene was located on rice chromosome 2: *xa24*.

This gene is a recessive gene against rice BB. This gene was identified in DV86 and then confirmed as a new gene. The gene was resistant to Filipino races 4, 6, 10, and Chinese strains Zhe173, JL691, and KS-1-21 during the whole growth period and was mapped between SSR markers RM14222 and RM14226 with a genetic distance of 0.14 cM and a physical distance of 71 kb (Khush and Angeles, 1999; Wu et al., 2008a).

#### Rice Chromosome 3

Two resistance genes were mapped on rice chromosome 3: *Xa11* and *xa42*.

*Xa11*, a dominant gene resistant to rice BB, is derived from IRBB11 (in the genetic background of IR24), resistant to Japanese races IB, II, IIIA, and V. The gene was located between markers RM347 and KUX11 at the short arm of chromosome 3, and the genetic distances from the two markers were 2.0 and 1.0 cM, respectively (Goto et al., 2012).

*xa42*, a recessive gene resistant to BB of rice, was derived from the mutant XM14 of IR24 which was resistant to six Japanese races. The gene was identified by linkage analysis around the chromosome 3 centromere region between DNA markers KGC3_16.1 and RM15189 (Constantine et al., 2016). After that, the gene was further narrowed to the 57 kb chromosome region between DNA markers KGC3_16.341 and KGC3_16.399 (Busungu et al., 2018). The gene was co-segregated with two DNA markers KGC3_16.370 and KGC3_16.371.

#### Rice Chromosome 4

Seven resistance genes were located on chromosome 4 of rice: *Xa1*, *Xa2*/*Xa31*, *Xa12*, *Xa14*, *Xa25*, *Xa38*, and *Xa45*.

*Xa1* is resistant to rice BB dominant gene, resistant to Japanese strains X-17, T7174, etc. The gene was identified from cultivated rice Kogyoku and Java14 and was located on chromosome 4 (Sakaguchi, 1967). The gene was subsequently found to be closely linked to three RFLP markers XNpb235, XNpb264, and C600 (Yoshimura et al., 1996).

*Xa2* is resistant to rice BB dominant gene, resistant to Japanese strains X-17, X-14, etc. The gene was located on chromosome 4 with a linkage rate of 2–16% with *Xa1*. The gene was fine mapped in the 190 kb region between markers HZR950-5 and HZR970-4 by SSR (He et al., 2006). *Xa31* is located in the 0.2 cM interval between G235 and C600 at the long arm end of chromosome 4 in rice, and the physical region of this interval in Minghui 63 is about 100 kb (Ogawa et al., 1978). *Xa2* and *Xa31* have the same sequence, which is actually the same gene.

*Xa12*, a dominant gene resistant to BB of rice, is derived from rice variety Java14 and resistant to Indonesian race V (Bao et al., 2006). This gene is located at the long arm end of chromosome 4 (Taura et al., 1992a). *Xa1* is closely linked to *Xa12*.

*Xa14*, a dominant gene resistant to rice BB, is resistant to race 4 in the Philippines. This gene is preliminarily located on chromosome 4 through rice trisomic technology (Tan et al., 1998). The F2 population was constructed by using susceptible pearl dwarf and near-isogenic line CBB14 containing *Xa14* as parents, and then, *Xa14* was located in the 0.21 cM interval between RG620 and G282 by RFLP analysis. By using IR24 × IRBB14 hybrid F2 to isolate 599 individuals, *Xa14* was finally finely mapped between molecular markers 52940 and RM5473 (Wang et al., 2009).

*Xa25*, a dominant gene resistant to rice BB, is derived from a somatic mutant HX3 and is resistant to Philippine races 1, 3, and 4. The gene was mapped between two SSR markers RM6748 and RM1153 at the end of the long arm of chromosome 4 by linkage analysis, and the linkage distances were 93 and 30 cM, respectively (Ying et al., 2002).

*Xa38*, a dominant gene resistant to BB of rice, was identified from *Oryza nivara* IRGC81825 and was resistant to all seven prevalent pathogenic *Xoo*s in northern India (Cheema et al., 2008). The gene was mapped within the genetic distance of 35 cM at the long arm of chromosome 4 through 191 polymorphic SSR markers and located between markers RM317 and RM562. Through further screening, the gene was finally located between marker RM17499 and STS markers based on annotated genes LOC_Os04g53060 and LOC_Os04g53120, about 38.4 kb distance.

*Xa45*, a dominant gene resistant to rice BB, is resistant to AXO1947 and T7174. The gene is an allele of *Xa1*, which is distributed on chromosome 4 and also encodes NLR-like proteins, and was successfully cloned recently (Biaoming et al., 2020).

#### Rice Chromosome 5

Only one resistance gene was located on chromosome 5 of rice: *xa5*.

This gene is a recessive gene for resistance to BB of rice. Three varieties DV85, DV86, and DZ78 from Bangladesh are resistant to PXO61 and PXO86. The gene was located between SNPS marker RS7 at the short arm end and SSR marker RM611, with a genetic distance of 0.5 cM and a physical distance of 70 kb (Blair et al., 2003).

#### Rice Chromosome 6

Four resistance genes were located on chromosome 6 of rice: *Xa7*, *Xa27*, *xa33*, and *xa42 (2)*.

*Xa7*, the dominant gene for resistance to BB of rice, was derived from three varieties DV85, DV86, and DZ78 from Bangladesh. It was resistant to PXO61, PXO86, PXO79, and SCB4-1 and was the dominant gene for resistance at booting stage. The gene was located between markers GDSSR02 and RM20593, and the genetic distances from markers were 0.07 and 0.14 cM, respectively. The gene was further locked in a range of 28 kb through map cloning and radiation mutagenesis (Chen et al., 2021).

*Xa27*, a dominant gene resistant to BB of rice, was derived from tetraploid small-grain wild rice and resistant to race 2, 5 of the Philippines. The gene was located in the range of 0.052 cM between RFLP markers M964 and M1197 and was co-segregated with RFLP markers M631, M1230, and M449. RGP markers C12560S and S12715 were of RFLP markers M964 and M1197, and the genetic distance between them was 0.9 cM (Gu et al., 2004).

*xa33*, a recessive gene resistant to BB of rice, was derived from the variety Ba7 and resistant to the Thai race TXO16. The gene was located between RM30 and RM400 by SSR marker-assisted selection. *Xa33* and *Xa7* are closely linked to SSR markers RM20590, but the resistance characteristics are significantly different from *Xa7* and *Xa27* (Kumar et al., 2012).

*xa42 (2)*, a recessive gene resistant to rice BB, was derived from a self-bred variety Baixiangzhan (BXZ). *xa42 (2)* showed broad-spectrum resistance to five *Xoo* pathogenic types in China, including the epidemic high-toxic *Xoo* pathogenic Chinese Race V (CV). The gene was mapped on chromosome 6 by linkage analysis and restricted to 3.9 cM region by genetic mapping, flanked by RM20558/RM20547 and RM20580 (Liang et al., 2017). The other seven markers were developed from this interval for high resolution mapping, and *xa42 (2)* was reduced to a 34.8 kb fragment defined by RM20572 and DT46.

#### Rice Chromosome 7

Two resistance genes were located on rice chromosome 7: *xa8* and *Xa33*.

*xa8* is a recessive gene resistant to rice BB, PI231129 from the United States, a strain resistant to northwestern India, with a genetic distance of 19.9 cM to marker RM214 (Sidhu et al., 1978). With the emergence of new SSR markers in the region, the gene was located between two continuous SSR markers, 7.0 and 9.9 cM, RM21044 and RM21045, respectively (Vikal et al., 2014).

*Xa33*, a dominant gene resistant to rice BB, was derived from *Oryza nivara* IRGC105710. The material showed broad-spectrum high resistance to BB and resistance to Indian race I-VII. The gene was initially mapped within 36.3 cM distance between RM5711 and RM6728 on chromosome 7 by 72 SSR markers. Subsequently, through the 2011 individuals of BC2F2 population, the gene was finally finely mapped within the genetic distance of 2.1 cM between RMWR7.1 and RMWR7.6 (Natraj et al., 2012).

#### Rice Chromosome 8

Only one resistance gene located on rice chromosome 8: *xa13*.

This gene is a recessive gene for resistance to BB of rice, derived from varieties BJ1 and DV85, and has specific resistance to race 6 of the Philippines (Ogawa, 1993). The gene was further mapped to a 9.2 kb fragment by recombination and a newly developed CAPS marker ST12 (Chu et al., 2006).

#### Rice Chromosome 11

Fifteen resistance genes were located on chromosome 11 of rice: *Xa3*/*Xa26*, *Xa4*, *Xa10*, *Xa21*, *Xa22*, *Xa23*, *Xa30*, *Xa32*, *Xa35*, *Xa36*, *Xa39*, *Xa40*, *xa41*, *Xa43*, and *xa44*.

*Xa3*/*Xa26*, *Xa22*, and *Xa4* were dominant genes resistant to rice BB and were located on M3H8 subclones at the end of the long arm of chromosome 11, between markers RM224 and RM114. The genetic distances between *Xa3*/*Xa26*, *Xa22*, and *Xa4* and RFLP marker R1056 were 0, 0.4, and 0.5 cM, respectively. Marker R1506 was located at 116.2 cM on chromosome clone OSJBa0047M04 (Chuntai et al., 2003; Yang et al., 2003). By analyzing the sequence of BAC clone 3H8, RKb gene was identified as *Xa26* gene (Sun et al., 2004). *Xa3*/*Xa26* was finely mapped on a 20 kb chromosome fragment of BAC clone M3H8 through the utilization of five double-exchange resistant plants of “Zhenshan 97”/“Minghui 63” recombinant inbred lines and finally cloned successfully in a BAC clone M3H8 of MH63 (Yi et al., 2006).

*Xa10*, a dominant gene resistant to rice BB, derived from cultivar Cas209, has specific resistance to four races in the Philippines (Yoshimura et al., 1983). The gene was located on chromosome 11, with a map distance of 5.3 cM from RAPD molecular marker O072000. Through linkage analysis of RFLP markers and F2 mapping population, *Xa10* was mapped to the proximal side of E1981S with a genetic distance of 0.93 cM (Gu et al., 2008). Based on five new RFLP markers developed from Nipponbare genome sequence, *Xa10* was fine mapped between the proximal marker M491 with a genetic distance of 0.28 cM and the distal marker M419 with a physical distance of 74 kb. Finally, *Xa10* was successfully map cloned (Dongsheng et al., 2014).

*Xa21*, a dominant gene resistant to rice BB, was derived from long-drug wild rice, resistant to races 1–6 in the Philippines. *Xa21* gene was located in the 8.5 cM interval on chromosome 11 (Ronald et al., 1992). At the same time, *Xa21* gene was co-segregated with molecular markers such as RG103, RAPD818, and RAPD248. By using the molecular marker RG103 as a probe to screen the 16 overlapping clones, *Xa21* gene was identified in a 9.6 kb fragment (Song et al., 1995).

*Xa23* is a dominant gene resistant to rice BB. *Xa23* gene has a wide spectrum of resistance. There are 20 domestic and foreign BB identification strains resistant to Philippine race 1–10, Chinese pathogenic race 1–7, and Japanese race 1–3. *Xa23* was identified in 1998 and then transferred to “Jingang 30” to develop a near-isogenic line CBB23 (Li et al., 2006). By using the F2 generation of hybrid between “Jingang 30” and CBB23 as the mapping population, molecular markers closely linked to *Xa23* were searched, BAC library and Shotgun library were constructed, and the corresponding sequencing and genetic transformation were carried out. Finally, *Xa23* gene was successfully cloned (Chunlian, 2006). The gene is located between markers C189 and RM206, and the genetic distances are 0.8 and 1.9 cM, respectively. The genetic distance from the RFLP marker C1003A on the same side of C189 is 0.4 cM. The markers C1003A and RM206 cover five clones: OSJNBa0072L08, OSJNBa006K21, P0480H08, OSJNBa0029K08, and B1356F10, and the genetic position is 84.6–88.4 cM.

*Xa30*, a dominant gene resistant to rice BB, is derived from common wild rice variety Y238 and is resistant to PXO99 (Philippine race 6). Four markers RM1341, V88, C189, and STS03 that reveal resistance and susceptibility polymorphism were screened from 343 molecular markers and then used for molecular detection and linkage analysis of 303 individuals in BC6F2 population. The results showed that the above four markers were all located in the long arm of chromosome 11 of rice, and the genetic distances with *Xa30* were 11.4, 11.4, 4.4, and 2.0 cM, respectively, and they were located on the same side of *Xa30* gene (Jin et al., 2007). The genetic distance between *Xa30* and marker STS03 was 2.0 cM, closer to the end of long arm than *Xa23*, and the genetic distance between RM206 and RM224 was more than 10 cM. Therefore, the position of *Xa30* on chromosome was closer to centromere than that of *Xa3*/*Xa26*.

*Xa32* (Zheng et al., 2009), *Xa35* (Guo et al., 2010), and *Xa36* (Miao et al., 2010) were all dominant genes for resistance to BB of rice. *Xa32* comes from C4064, resistant to Pr I, IV-IX, and KXO85. *Xa35* is derived from Acc. No.101133, resistant to PXO61, PXO112, and PXO339. *Xa36* comes from C4059, resistant to PXO99. The three were all located at the long arm end of chromosome 11. The genetic distances between *Xa4* and *Xa36* and the marker RM224 were 1.1 and 1.3 cM, respectively. It was speculated that *Xa36* was closer to the long arm end than *Xa4*. *Xa35* was co-segregated with the marker RM114, closest to the long arm end. The genetic distances between *Xa32* and *Xa36* and the marker RM5926 were 2.6 and 3.8 cM, respectively. It was speculated that *Xa32* was between *Xa36* and *Xa35*. Five pairs of polymorphic molecular markers were newly developed between resistant material Q143 and susceptible parent JG30, which were K58, K57, Y72FF, Y77, and Y99, respectively (Zheng et al., 2009). These five polymorphic markers were closely linked to the *Xa32* gene, and *Xa32* was located within 0.16 cM between the end marker Y72FF of the long arm of chromosome 11 and the marker Y77 of rice. Then, the physical map of *Xa32* gene locus was constructed, and *Xa32* gene was located within the range of about 70 kb at the long arm end of rice chromosome 11.

*Xa39*, a dominant gene resistant to BB of rice, was derived from a rice introgression line FF329, which was screened from BC1F4, a backcross progeny of PSBRC66 (P66) and Huang Huazhan (HHZ). *Xa39* was located at a distance of 97.4 kb between RM26985 and DM13 on chromosome 11 (Zhang et al., 2015).

*Xa40*, a dominant resistance gene to rice BB, is a new resistance gene identified from indica rice IR65482-7-216-1-2, showing high level resistance to all Korean *Xoo* races, including the new *Xoo* race K3a (Kim et al., 2015). The gene was mapped to an 80 kb region between RM27320 and ID55.WA18-5 on chromosome 11, which contains eight predicted candidate genes.

*xa41*, a recessive gene resistant to rice BB, derived from *Oryza barthii* and *Oryza glaberrima*, is resistant to BAI3. After screening the polymorphism of 169 rice germplasms in the promoter of the susceptible gene *OsSWEET14* for BB, the gene was found (Hutin et al., 2016). The deletion of *OsSWEET14* gene promoter region18 bp represents a new resistance allele, namely *xa41*.

*Xa43*, a dominant gene resistant to rice BB, derived from P8, is resistant to Korean race HP01009. The target region was dissected by QTL analysis and fine mapping, and the mapping region of this gene was narrowed to 27.83–27.95 Mbp of PCR-based DNA markers IBb27os11_14 and S_BB11. ssr_9, with a physical interval of about 119 kb (Kim et al., 2019).

*xa44*, a recessive gene resistant to rice BB, derived from P6, is resistant to the Korean race HB1009. Due to the additional development based on PCR markers in the target region, the target region of SNP flanking (28.00–28.12 Mbp) was further narrowed. Through fine mapping of 520 BC1F2 individuals, R gene *xa44* was identified in a fragment of about 120 kb, with DNA markers # 46. g0689400 and RM27318 on both sides (Kim, 2018).

#### Rice Chromosome 12

Two resistance genes were located on rice chromosome 12: *xa25* and *xa32*.

*xa25*, a recessive gene resistant to BB of rice, is derived from Minghui 63 and has transformation resistance to Philippine race 9 (PXO339) at seedling and adult stages. This gene is located at 9.5 cM between RFLP markers R887 and G1314 on chromosome 12 of rice (Chen et al., 2002).

*xa32*, a recessive gene resistant to BB of rice, is a new germplasm Y76 derived from the somatic hybridization between wild wart grain rice and cultivated rice in Xishuangbanna, Yunnan. It is resistant to PXO99 (race 6 of the Philippines), and the genetic distance from the marker RM20A is 1.7 cM (Ruan et al., 2008).

In summary, the basic information of 44 rice BB resistance genes that have been mapped can be found in Table 1.

**Table 1:** The identified rice bacterial blight (BB) resistance genes.

Gene	Dominance/recessive	Strain(race)	Linkage labeling	Chromosome
*Xa1*	+	T7174	C600 (0 cM), XNpb235 (0 cM), U08750 (1.5 cM)	4
*Xa2*/*Xa31*	+	T7174, OS105	HZR950-5 ~ HZR970-4 (190 kb), G235 ~ C600 (0.2 cM)	4
*Xa3*/*Xa26*	+	PXO61, PXO86	XNbp181 (2.3 cM), RM224 (0.21 cM), Y6855R (1.47 cM)	11
*Xa4*	+	PXO61, PXO71, and PXO112	XNpb181 (1.7 cM), XNpb78 (1.7 cM)	11
*xa5*	−	PXO61, PXO86	RS7 ~ RM611 (0.5 cM)	5
*Xa7*	+	PXO61, PXO86, PXO79, and SCB4-1	M5 ~ M10 (28 kb)	6
*xa8*	−	Ir PbXo-I, II, III, IV, and VI	RM21044 (7.0 cM) ~ RM21045 (9.9 cM)	7
*Xa10*	+	PXO99A	O072000 (5.3 cM), M491 ~ M419 (0.28 cM)	11
*Xa11*	+	T7156, T7147, T7133, and H75304	RM347 (2.0 cM), KUX11 (1.0 cM)	3
*Xa12*	+	Inr V		4
*xa13*	−	PXO99	RZ28 (5.1 cM), G136 (3.8 cM), RP7 ~ ST12 (9.2 kb)	8
*Xa14*	+	PXO112	RG620 ~ G282 (0.21 cM)	4
*xa15*	−	Jr I, II, and III		—
*Xa16*	+	H8584		—
*Xa17*	+	PXO99		—
*Xa18*	+	BM8417, BM8429		—
*xa19*	−	Pr I-VI		—
*xa20*	−	Pr I-VI, Jr I-IV		—
*Xa21*	+	Pr I, II, IV, and VI	RG103 (0 cM)	11
*Xa22*	+	PXO61	CR543 (7.1 cM), RZ536 (10.7 cM), Y6855RA (0.4 cM), G2132B (0.7 cM)	11
*Xa23*	+	PXO99	C189 (0.8 cM), CP02662 (1.3 cM)	11
*xa24*	−	PXO61, PXO86, PXO71, PXO99, and JL691	RM14222 ~ RM14224 (10 kb)	2
*xa25*	−	PXO339	G1314 (7.3 cM), R887 (3.0 cM)MZ2 (0.38 cM), MZ7 (0.06 cM)	12
*Xa25*	+	Zhe173	RM6748 (9.3 cM), RM1153 (3.0 cM)	4
*Xa27*	+	Pr II, V, and VI	M964 ~ M1197 (0.052 cM)	6
*xa28*	−	Pr II		—
*Xa29*	+	PXO61	C904 ~ R596 (1.3 cM)	1
*Xa30*	+	PXO99	03STS (2.0 cM)	11
*Xa32*	+	Pr I, IV-IX, and KXO85	ZCK24 (0.5 cM) ~ RM6293 (1.5 cM), Y72FF ~ Y77 (0.16 cM)	11
*xa32*	−	PXO99	RM20A (1.7 cM)	12
*xa33*	−	TXO16	RM30 ~ RM400	6
*Xa33*	+	Ir I-VII	RMWR7.1 (0.9 cM) ~ RMWR7.6 (1.2 cM)	7
*xa34*	−	5226	RM10929 ~ BGID25 (204 kb), RM10926 ~ InDelnv7 (56.47 kb)	1
*Xa35*	+	PXO61, PXO112, and PXO339	RM7654 (1.1 cM) ~ RM6293 (0.7 cM)	11
*Xa36*	+	PXO99, C5	RM224 ~ RM2136 (4.5 cM)	11
*Xa38*	+	Ir I-VII	RM17499 (38.4 kb)	4
*Xa39*	+	Pr I-X, Cr II, and IV-VII	RM26985 ~ DM13 (97.4 kb)	11
*Xa40*	+	HB01013, HB01014, HB01015, and HB01009	RM27320 ~ ID55. WA18-5 (80 kb)	11
*xa41*	−	BAI3	RM27320 ~ RM27335	11
*xa42*	−	T7174, T7147, T9387, T7133, H75373, and H75304	KGC3_16.341 ~ KGC3_16.399 (57 kb)	3
*xa42(2)*	−	SCB4-1	RM20572 ~ DT46 (34.8 kb)	6
*Xa43*	+	Kr HP01009	IBb27os11_14 ~ S_BB11.ssr_9	11
*xa44*	−	Kr HB1009	#46.g0689400 ~ RM27318 (120 kb)	11
*Xa45*	+	AXO1947, T7174		4

Annotation 1. Ir, Indian race; Inr, Indonesian race; Jr, Japanese race; Pr, Philippine race; and Kr, Korean race.

## Cloning and Function Research Progress of Rice BB Resistance Gene

**Figure 2:**
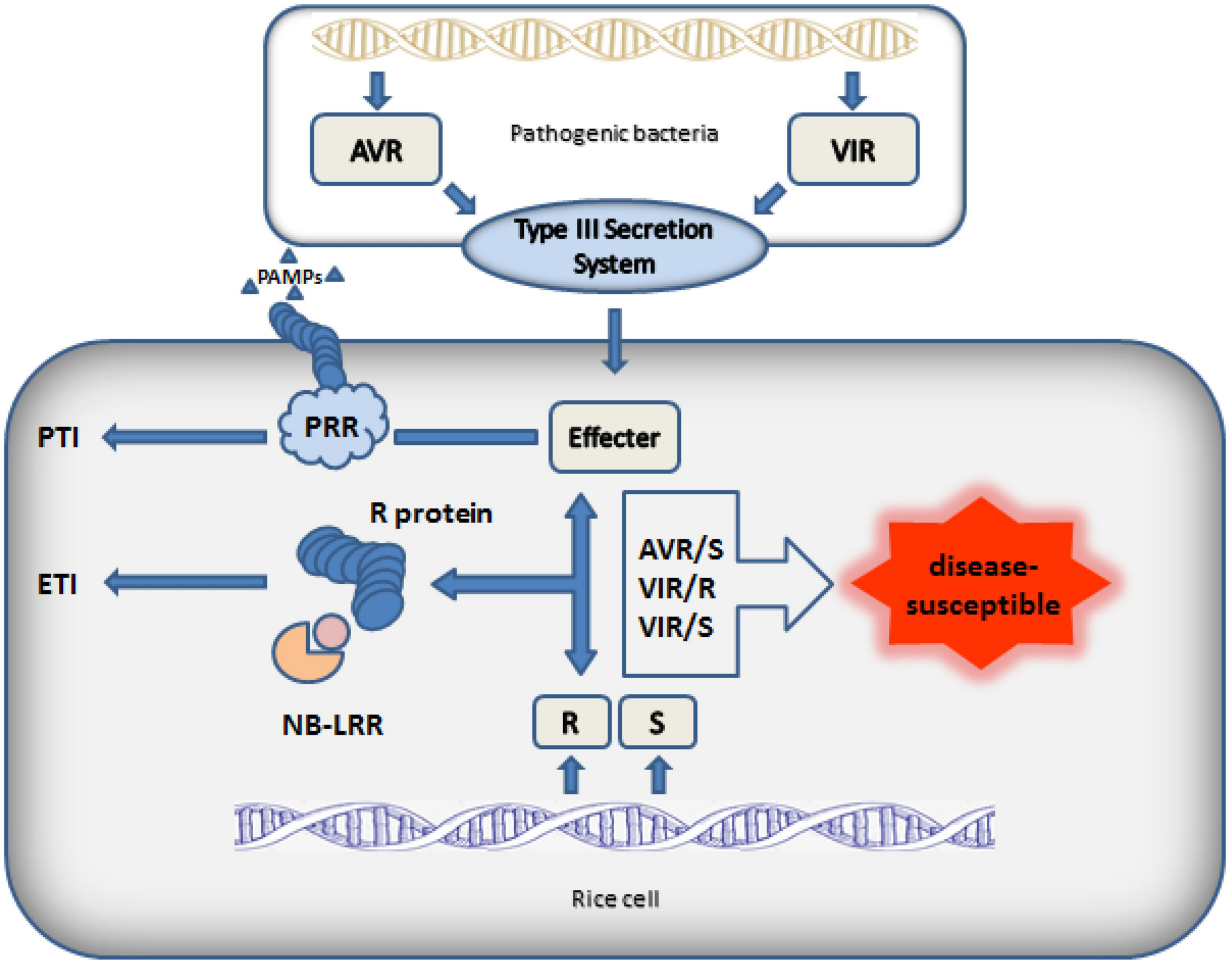
Interaction model between pathogen and rice.

After breaking through the first line of defense PTI, pathogenic microorganisms evolved effector proteins to inhibit the immune response caused by pathogen associated molecule patterns (PAMPs) in order to successfully infect plants. At the same time, plants have evolved R genes to recognize effectors and cause hypersensitive reaction of cells. Only when the avirulence genes of the pathogen and the R genes of the host cell exist at the same time, the plant disease resistance gene can play its own disease resistance function (Figure 2). Therefore, after gene mapping, people continue to clone new rice R genes to resist the infection of new avirulence genes. Next, the cloning and function of rice BB resistance gene will be described in detail.

### Xa1

*Xa1* gene is the first reported NLR resistance genes in BB resistance gene. The gene was located in a YAC clone by DNA hybridization and recombination events and then isolated by map cloning (Yoshimura et al., 1996). But unlike the strategy of cloning *Xa21*, *Xa1* was cloned by cDNA (Yoshimura et al., 1998). The gene contains four exons and three introns, one of which has no coding sequence. The gene encoding product is a cytoplasmic protein composed of 1,802 amino acids. Its amino end contains the NBS domain, and the carboxyl end contains the LRR functional domain, that is, the NBS-LRR-type resistance protein to BB (Yoshimura et al., 1996).

*Xa1* is highly resistant to type I physiological race of BB in Japan and has specific resistance. Different from other resistance genes, the expression of *Xa1* is induced by pathogen infection and trauma rather than constitutive expression. Studies have shown that the expression of *Xa1* is induced by both compatible physiological races and incompatible physiological races, indicating that the damage caused by pathogen infection may induce the expression of *Xa1* gene, and the increase in *Xa1* gene expression may improve the efficiency of *Xa1* interacting with an avirulence gene, thereby activating the *Xa1*-mediated disease resistance signaling pathway, so *Xa1* plays a major role in the identification of pathogens (Ji et al., 2020). Recently, *Xa2*, *Xa14*, *Xa31*, and *Xa45* have also been successfully cloned. These four genes, as alleles of *Xa1*, also encode NLR proteins (Ji et al., 2020; Zhang et al., 2020).

### Xa3/Xa26

*Xa3* was located in IRBB13, and *Xa26* gene was identified in rice cultivar “Minghui 63.” After DNA fingerprinting analysis, fine mapping, phenotypic comparison after inoculation with *Xoo* and sequence analysis of candidate genes, these two genes were found to be the same gene and renamed as *Xa3*/*Xa26* (Yoshimura et al., 1995; Peng et al., 1998; Yi et al., 2006). The *Xa3*/*Xa26* gene was constitutively expressed. The coding region was 3,309 bp in length, with two exons and a 105 bp intron located on the cell membrane. The gene encodes a LRR receptor protein kinase composed of 1,103 amino acids, which belongs to the same type as *Xa21*. The structural characteristics of the two genes are very similar, including an amino-terminal signal peptide composed of 30 amino acids, an extracellular LRR domain composed of 26 incomplete LRRs, a single transmembrane region (TM), and an intracellular protein serine/threonine kinase domain (Sun et al., 2004). The amino acid of 88 ~ 771 encodes 26 incomplete leucine-rich repeats (LLRs), which are conserved sequences encoded by these genes. The main function is to identify specific pathogens and then stimulate disease resistance through signal transduction (Song et al., 1995).

*Xa3*/*Xa26* belongs to LRR-STK resistance type and is a multi-gene family, including four members: RKa, RKb, RKc, and RKd. The gene is clustered and arranged on chromosome 11 with high conservation (Wang, 2015). It was found that the *Xa3*/*Xa26* LRR domain may be involved in the identification of different *Xoo* races (Jing et al., 2009). *Xa3*/*Xa26* is only specifically expressed in vascular bundles and surrounding cells and is low-level constitutive expression (Cao et al., 2007). The infection of pathogens had no obvious effect on the transcriptional expression intensity (Sun et al., 2004). The resistance of *Xa3*/*Xa26* was affected by the growth period of rice. The rice containing *Xa3*/*Xa26* was susceptible to PXO61 at seedling stage and resistant to PXO61 at adult stage. This was due to the dose effect of *Xa3*/*Xa26*. The expression level of *Xa3*/*Xa26* was very low at the two-leaf stage and reached the highest at the tillering stage. The overexpression of *Xa3*/*Xa26* at the seedling stage showed resistance to BB of rice. The resistance of *Xa3*/*Xa26* is also affected by genetic background. Studies have shown that the broad and narrow resistance spectrum and the strength of resistance of *Xa3* are significantly different under different genetic backgrounds, and even there is a phenomenon of recessive transformation, which is also the reason for the diversification of *Xa3* gene naming (*Xa4b*, *Xa6*, and *xa9*). The resistance is different in different indica rice and japonica rice, but in general, the resistance of *Xa3*/*Xa26* in japonica rice is stronger than that in indica rice (Cao et al., 2007). The *Xa3*/*Xa26* gene family reflects the competition and co-evolution of rice BB resistance genes and pathogens. Some genes have been resistant to BB and then lost resistance. However, in the later evolution process, they have regained the function of disease resistance by point mutation (Cao et al., 2007).

### Xa4

*Xa4* was located on the M3H8 subclone at the end of the long arm of chromosome 11 and located between markers RM224 and RM114 (Lee et al., 2003). The genetic distance between *Xa4* and RFLP marker R1056 was 0.5 cM, and the marker R1506 was located at 116.2 cM on the chromosome clone OSJBa0047M04. Through recombinant test and sequence analysis, the *Xa4* gene was fine mapped within 47Kb (Sun et al., 2003) and then successfully cloned. *Xa4* encodes a wall-associated kinase (WAK) consisting of 707 amino acids, containing a predicted galacturonic acid binding domain, a calcium-binding epidermal growth factor domain, a transmembrane helix, and a STK domain. The protein encoded by recessive *xa4* has an amino acid variation (D152E) after the galacturonic acid binding domain (Keming et al., 2017).

*Xa4* is a dominant gene, which was identified in cultivated rice varieties IR20, IR22, IR72, and TKM6. *Xa4* is mainly resistant to Philippine races 1, 5, 7, and 8 and also has strong resistance to seven BB races in China (Amante-Bordeos et al., 1992). The cell WAK encoded by *Xa4* can accelerate the synthesis of cell cellulose, thereby inhibiting cell wall porosity, strengthening plant cell wall, and effectively preventing the invasion of pathogens. It is a natural barrier for plant protection. In addition, *Xa4* can achieve excellent improvement of plant height without affecting the yield of grain crops. By reducing the height of the plant and improving the mechanical strength of the plant, the plant is not easy to break under harsh natural conditions, which further improves the lodging resistance of rice plants and is conducive to practical production and utilization. Due to the simultaneous improvement of multiple key agronomic traits, *Xa4* has been widely used in the global rice breeding program (Keming et al., 2017).

### xa5

*xa5* was derived from three Bangladesh varieties DV85, DV86, and DZ78 and then was initially located on chromosome 5 (Iyer and Mccouch, 2004). By using near-isogenic lines IRBB5 and IR24 as parents, *xa5* was finely mapped between SNPS marker RS7 and SSR marker RM611 at the short arm end of chromosome 5, with genetic distance of 0.5 cM and physical distance of 70 kb (Blair et al., 2003). Different from the above functional complementation verification methods of cloned genes, the confirmation of candidate genes of *xa5* was performed by mutant analysis, and then cloned successfully (Iyer and Mccouch, 2004). Chinese researchers have continued to complete the functional complementation test of *xa5* candidate genes, which further confirmed the previous results (Jiang et al., 2006).

*xa5* is an incomplete recessive gene for resistance during the whole growth period, which is constitutive expression like its allelic dominant gene *Xa5* (Jiang et al., 2006). The coding product of this gene is the γ subunit of transcription factor TFIIA, which is composed of 106 amino acids. The corresponding DNA sequence contains four exons (one of which has no coding sequence) and three introns. TFIIAγ is a transcription factor of eukaryotes. It has 50% homology with TFIIAγ of Drosophila and human, and 85–93% homology with *Arabidopsis*, maize, barley, sugarcane, and wheat. Most plants and rice have the same TFIIAγ susceptibility alleles.

*xa5* is resistant to races 1, 2, 3, and 5 of the Philippines (Li et al., 1999). *xa5* can inhibit the transfer of *Xoo* invading rice, thereby resisting BB of rice (Iyer-Pascuzzi et al., 2008). In addition, this gene has a typical structure of disease resistance gene, and glutamic acid is the 39th amino acid of its coding product. If the *xa5* coding sequence is mutated here, the expression product cannot interact with non-toxic effectors, and rice also loses its disease resistance function (Jiang et al., 2006). Therefore, the disease resistance of this gene is due to the change of two bases, which makes the encoded hydrophobic valine become hydrophilic glutamic acid. There are two pathogenic variants of *Xoo*, which are effectors (TALEs) of *Xoo* and *Alternaria solani*. They can directly interact with TFIIAγ5 and activate the expression of susceptible genes in the host. The mutation of recessive gene *xa5* destroys its combination with TALE, thus triggering plant defense responses (Yuan et al., 2016).

### Xa7

*Xa7*, a dominant gene resistant to rice BB, is a dominant gene resistant to rice BB at booting stage in three varieties DV85, DV86, and DZ78 from Bangladesh (Sidhu et al., 1978). It is located between markers GDSSR02 and RM20593, with genetic distances of 0.07 and 0.14 cM, respectively. In addition, the variety Zhenhui 084 of *Xa7* was also bred. After identification, *Xa7* has resistance to multiple physiological races of the Philippines in the whole growth period, which is of great significance for rice resistance to BB (Porter et al., 2003). *Xa7* was initially located at 107.5 cM on the long arm of chromosome 6 in rice, and then, the genetic map constructed by molecular markers was finely mapped. Through a series of polymorphic molecular markers such as AFLP, STS, and SSR, *Xa7* was mapped between M1 and M3 with a genetic distance of 2.7 cM (Porter et al., 2003). Under the utilization of more susceptible F2 plants of IR24 × IRBB7, *Xa7* was located in the region of 118.5 kb (Nipponbare genome; Shen et al., 2008). But the gene was not cloned for a long time. Recently, the cloning of *Xa7* gene has made breakthrough progress. At the same time, *Xa7* was mutated by radiation. Nine lines with high susceptibility to BB were screened from more than 20,000 mutant lines over 5 years. One mutant line happened to have chromosome deletion at the location of *Xa7* gene. The size of the deletion fragment was determined to be 107 kb by high-throughput sequencing. By comparing the overlapping regions between the gene localization region and the chromosome deletion region, *Xa7* was further locked in the 28 kb region. Through a large number of verification of transgenic functions of rice, *Xa7* was finally cloned successfully (Chen et al., 2021).

Studies have shown that *Xoo*’s two important TALEs (AvrXa7 and PthXo3) have overlapping regions in *Xa7* promoter effector binding elements (EBEs). *Xa7* is induced by PXO86 (avrXa7) and PXO61 (pthXo3) at the same time. AvrXa7 and PthXo3 can trigger *Xa7* expression, which makes rice materials containing the gene have broad-spectrum characteristics (Chen et al., 2021). In addition, by using IRBB7, IRBB4, and IRBB21 as donors, the polymeric resistance restorer lines with *Xa4* + *Xa7* double genes and *Xa4* + *Xa7* + *Xa21* triple genes were obtained, which provided the possibility for breeding new resistant varieties.

It was found that after more than 10 years of field planting, the resistance of *Xa7* gene could be maintained for more than 10 years (Cruz et al., 2000). Related studies have shown that the resistance of *Xa7* to BB is stronger and more durable than that of genes such as *Xa4* and *Xa10* (Webb et al., 2010). In general, high temperature is conducive to bacterial growth and promotion of infection. However, under the environmental conditions of high-temperature stress, the resistance pathway mediated by disease resistance gene *Xa7* can more effectively limit the growth and reproduction of *Beauveria bassiana* compared with genes such as *Xa3*, *Xa4*, *Xa5*, and *Xa10* (Cohen et al., 2017). RNA-Seq analysis showed that in susceptible plants, high temperature upregulated genes in plant hormone abscisic acid biosynthesis and response pathways, while in plants with *Xa7*-mediated resistance, high temperature inhibited the expression of these genes. Moreover, salicylic acid is an important plant disease resistance hormone in plant disease resistance response. Under high-temperature stress, the response genes of both resistant and susceptible plants to salicylic acid have been downregulated, indicating that the resistance enhancement mediated by *Xa7* under high temperature may not depend on salicylic acid signal transduction. This may be the reason why the resistance of resistance gene *Xa7* is more durable (Cohen et al., 2017). Other studies have shown that *Xa7* is induced by the secretion effector (TALEs) AvrXa7 of *Xoo* to produce defense responses to prevent the invasion of *Xanthomonas oryzae*. This induction is faster and stronger at high temperatures, and *Xa7* is more resistant.

### Xa10

*Xa10* was originally found in Cas209 and has race-specific resistance to BB of rice (Yoshimura et al., 1983). *Xa10* was initially located on the long arm of chromosome 11 of rice, in the 0.28 cM interval between markers M491 and M419, and was co-segregated with S723 and M604 (Gu et al., 2008). Then, the gene was cloned by map. *Xa10* encodes a small molecule protein composed of 126 amino acid residues, and it is predicted that the gene contains four potential transmembrane helixes and an ED domain at the C-terminal. The promoter region of *Xa10* has elements that bind to AvrXa10, and its transcription is specifically activated by AvrXa10 (Dongsheng et al., 2014).

The protein encoded by *Xa10* is located on the endoplasmic reticulum membrane in the form of hexaploid. It causes the hypersensitivity of host cells by affecting the balance of endoplasmic reticulum and intracellular calcium ions, leading to cell death, thereby limiting the continued reproduction and infection of pathogens, thereby producing BB resistance. The expression of *Xa10* in rice, tobacco, and salad cells leads to programmed cell death. It is speculated that *Xa10* is at the end of a signal pathway that can be induced to trigger programmed cell death, and this is achieved through a conservative system that maintains endoplasmic reticulum and Ca^2+^ homeostasis. In the F2 population of IR24 × IRBB10, it was found that *Xa10*-mediated resistance to PXO99A did not follow Mendelian genetic law; that is, the segregation ratio was not 3:1 (Gu et al., 2008). The new near-isogenic line IRBB10A of *Xa10* was developed under the genetic background of IR24. Its expression was induced by physiological race PXO99A, but it was not induced by uninoculated or inoculated with PXO99A without avrXa10, indicating that AvrXa10 could specifically recognize EBEAvrXa10 and initiate the expression of *Xa10*.

### xa13

*xa13* was located on the long arm of chromosome 8, and the genetic distance was less than 0.4 cM by using the F2 line of the hybrid offspring of IR24 × IRBB13 and the F2 line of the hybrid offspring of IRBB13 × IR65598-112 (Sanchez et al., 1999). Subsequently, *xa13* was successfully cloned through fine mapping and candidate target gene analysis. *xa13* is a recessive disease resistance gene, which has specific resistance to PXO99. *xa13* is composed of five exons, encoding SWEET protein containing 307 amino acids, which is located on the plasma membrane (Vikal et al., 2014).

Different promoter regions of *xa13* lead to differences in protein products encoded by its dominant allele *Xa13* gene (Ginny et al., 2010). *xa13* was hardly induced by pathogens and injuries, and its dominant allele *Xa13* could be strongly induced by PXO99 but not by mechanical injuries. It is believed that *xa13* is different from other disease-resistant genes, and its disease-resistant function is caused by the loss of the power of induced transcription caused by natural selection (Yuan et al., 2009). Both *xa13* and its dominant allele *Xa13* were expressed at low levels in rice leaves, but at high levels in inflorescence and pollen sac of rice. Studies have found that inhibiting the expression of *xa13* and *Xa13* at the same time will improve the resistance of plants, but will lead to male sterility in rice, indicating that *xa13* gene controls the disease resistance of rice and the growth and development of pollen (Li et al., 2012).

Copper is not only an important trace element in plants but also an important component of some pesticides. It can inhibit the growth of BB, and BB strain PXO99 is more sensitive to copper than other strains, which is also the reason why *xa13* has specific resistance to PXO99. The corresponding dominant gene *Xa13* of *xa13* is a susceptible gene, which is basically consistent with the coding region of *xa13*, and is different from the mutation of 18 bases in its promoter region. Because of the mutation of the cis-acting element of the promoter, PthXo1 secreted by the pathogen cannot bind to its promoter. PthXo1 cannot bind to the promoter of *xa13* gene and thus cannot induce the expression of *xa13* gene. Thus, plant host cells can inhibit the growth of PXO99A by maintaining the balance of copper ion concentration in vascular bundle ducts, thereby making plants resistant. In rice containing *Xa13*, copper transporters can play a role in the xylem of plants by binding to the coding product of *Xa13*, removing copper ions that can inhibit the pathogenic function of *Xoo*, leading to the rapid growth and reproduction of *Xoo* in xylem, and rice is susceptible (Yuan et al., 2010; Yuan and Wang, 2013).

### Xa21

*Xa21* is the first resistance gene isolated by map-based cloning, showing excellent resistance to physiological races 1–6 of BB in the Philippines (Song et al., 1995). This gene has been transferred into near-isogenic lines with IR24 as the background and has been widely used in rice genetic breeding. It was also found that *Xa21* gene was co-segregated with molecular markers such as RG103, RAPD818, and RAPD248. The *Xa21* gene was located and cloned by map-based cloning, but the homologous sequence method was also used in the study. The efficiency of cloning gene was greatly improved by using this method (Wang, 1996).

The gene contains an 843 bp intron that encodes a protein composed of 1,025 amino acids, and the encoded protein belongs to the receptor-like kinase (RLK; Song et al., 1995). The protein structure is divided into nine regions, including signal peptide region, unknown function region, LRRs, a charged region, TM, a charged region, perimembrane region (JM), serine–threonine kinase domain (STK), and carboxy-terminal tail region. The resistance of *Xa21* is related to the LRRs and STK regions. The resistance mediated by *Xa21* depends on the activity of these two regions. The former is composed of 23 incomplete LRRs, which are involved in protein–protein interactions and are related to pathogen identification (Wang et al., 1998). The latter is a typical signal molecule for early defense immune response of plants (Park et al., 2010).

It was found that the downstream signal transduction was determined by the kinase region and was transcribed throughout the whole process without the influence of *Xoo* infection. However, its disease resistance was affected by various stages of rice growth and development. With the growth and development of rice, its disease resistance gradually increased, and it was completely resistant at tillering stage (Zhang et al., 2010). The disease resistance of *Xa21* is similar to that of *Xa3*/*Xa26* and is also affected by genetic background (Jing et al., 2009). Under the background of “IR24” or “Minghui 63,” the resistance of *Xa21* and *Xa3*/*Xa26* to both compatible and non-compatible races gradually increased from seedling stage to adult stage, and the resistance change was the most obvious during the four-leaf stage to the peak tillering stage. However, under the background of “Mudanjiang 8,” *Xa21* and *Xa3*/*Xa26* resistance was not affected by growth period.

With the deepening of research, more and more *Xa21* binding proteins (XB) have been found to be involved in *Xa21*-mediated disease resistance (Pruitt et al., 2015). For example, XB3 is a positive regulator of XA21-mediated immune response. The combination of XA21 and XB3 will promote the phosphorylation of XB3 and stimulate disease resistance (Xu et al., 2006). XB10 is a negative regulator of XA21-mediated immune response, which can inhibit the expression of defense genes by binding to XA21 (Park et al., 2017). XB15 is a PP2C protein phosphatase that can negatively regulate cell death and negatively regulate XA21-mediated immune response (Wang et al., 2007). XB24 is an ATP enzyme that can promote the phosphorylation of XA21 (Ying et al., 2010). The autophosphorylation of Ser686, Thr688, and Ser689 can stabilize XA21 to resist development-controlled protease activity (Peng et al., 2008).

The avirulence gene AvrXa21 of XA21 is named RaxX. When the pathogen invades, the LRR region of XA21 protein recognizes and binds to the effector RaxX protein of the pathogen, thereby initiating the PTI process (Park et al., 2008). The specific interaction process between Xa21 and RaxX is that endoplasmic reticulum molecular chaperones and auxiliary molecular chaperones participate in the biosynthesis of Xa21. After the synthesis of Xa21 polypeptide, it needs to be processed by endoplasmic reticulum and transported to plasma membrane before it can play its biological function. When XB24 is combined with the intracellular near membrane region of XA21, XA21 is in the inactive state of self-phosphorylation. When RaxX binds to the extracellular LRRs domain of XA21, XB24 separates from Xa21 and activates the kinase domain of XA21. The self-phosphorylated Thr705 transfers the phosphoryl group to another residue of XA21, which activates XA21. Then, the XA21 kinase domain is segmented and transported to the cell core, which binds to the transcription factor (TF) WRKY62 and further triggers the expression of defense genes (Chen et al., 2010).

### Xa23

A new gene resistant to BB from common wild rice was identified by resistance spectrum evaluation, resistance-type comparison, and genetic analysis, which was named *Xa23* (Li et al., 2006). *Xa23* gene was transferred to “Jingang 30” and cultivated into near-isogenic line CBB23. The F2 generation of “Jingang 30” and CBB23 hybrids was used as mapping population, and then, the molecular markers closely linked to *Xa23* were found through various ways. BAC library and Shotgun library were constructed for sequencing and genetic transformation. Finally, *Xa23* gene was successfully cloned (Chunlian, 2006). *Xa23* was located between markers C189 and RM206, with a genetic distance of 0.8 and 1.9 cM, respectively. The genetic distance of RFLP marker C1003A from the same side of C189 was 0.4 cM. Markers C1003A and RM206 covered five clones: OSJNBa0072L08, OSJNBa006K21, P0480H08, OSJNBa0029K08, and B1356F10, with a genetic position of 84.6–88.4 cM.

*Xa23* gene has a broad spectrum of resistance, resistance to Philippine races 1 ~ 10, Chinese pathogenic races 1 ~ 7, and Japanese races 1 ~ 3, a total of 20 domestic and foreign BB identification strains, and completely dominant and whole growth resistance (Wang et al., 2014b). Through the identification of *Xa23* transgenic plants resistance to BB, it was found that when the *Xa23* gene insertion site was different, the degree of disease resistance was significantly different, and the degree of disease resistance of T0 plants can be accurately and stably inherited to T1 and T2 generations (Zhou et al., 2011). *Xa23* encodes a protein consisting of 113 amino acids, which has 50% homology with the known Executor protein XA10. The transmembrane helix structure of *Xa23* protein is also partially overlapped with that of *Xa10* protein. Unlike *Xa10*, *Xa23* has broad-spectrum resistance and can be activated by TALE effector AvrXa23 (Wang et al., 2015). *Xa23* transcription is specifically induced by AvrXa23. The susceptible xa23 allele has the same open reading frame region (ORF113) as the dominant gene *Xa23* for disease resistance. The reason for the polymorphism between the two genes is that the promoter region is different. The susceptible *xa23* lacks the TALE binding element (EBE) that can specifically bind to AvrXa23. Under normal conditions, the ORF113 of *Xa23* had a very low transcription level in resistant and susceptible varieties, but it was induced by pathogenic bacteria in CBB23 and transgenic plants. This induced expression was not detected in susceptible varieties JG30, Nipponbare, and MDJ8. ORF113-RNAi plants were susceptible to PXO99A, indicating that *Xa23*-mediated resistance needs to increase the expression of ORF113. There is a 7 bp polymorphism in the promoter region of resistant *Xa23* in susceptible *xa23* and CBB23 in JG30, which is related to the effector binding element of AvrXa23 (Wang et al., 2015). These results suggest that *Xa23* plays a role in disease resistance by identifying TALEs in pathogens.

### xa25

*xa25* is derived from the cultivated rice “Minghui 63.” The gene is located between RFLP markers R887 and G1314 on chromosome 12 of rice, with the map distances of 3.0 and 7.3 cM, respectively (Chen et al., 2002), and then isolated through map cloning (Liu et al., 2011). *xa25* has specific resistance to physiological race PXO339 and belongs to the sugar transporter (SWEET) family gene (Rmer et al., 2010). *xa25* encodes a MtN3/saliva family protein containing 296 amino acids, which is ubiquitous in eukaryotes. Its allelic dominant susceptibility gene *Xa25* also encodes a member of the MtN3 gene family, and the encoded product is a protein composed of 293 amino acids. The resistance mediated by *Xa25* is different from that of most other R genes. It shows recessive resistance at seedling stage and dominant resistance at adult stage (Liu et al., 2011). Not only the number of amino acids is different, but also the encoded protein has eight amino acids.

*xa25* showed unique resistance to PXO339 by inhibiting the growth of *Xoo* at seedling stage and mature stage, respectively. The transfer of *Xa25* to resistant rice carrying *xa25* could lead to the weakening of resistance to PXO339. Only PXO339 in different strains could rapidly induce the expression of *Xa25*, but did not induce the expression of *xa25*. It was speculated that *Xa25* and *Xa13* were also induced by specific TAL effectors in pathogens, thus causing host susceptibility. To confirm this hypothesis, promoter structure analysis showed that *xa25* from resistant varieties and *Xa25* from susceptible varieties had multiple sites in the promoter region, which may be the cause of different resistance (Liu et al., 2011).

The cis-acting element of dominant *Xa25* gene promoter contains PthXo2 binding site, which can be recognized and induced by effectors. *Xa25* is a sucrose transporter gene. Its expression can leak sucrose, glucose, and other photosynthetic products to the intercellular space of rice. These substances are used by pathogenic bacteria, which are beneficial to the infection and reproduction of pathogenic bacteria, and then make plants susceptible. However, there is a mutation at this site on the cis-acting element of the recessive *xa25* gene promoter, so that it could not be recognized by the pathogen effector PthXo2, and thus, the plant showed resistance to diseases (Zhou et al., 2015). Therefore, compared with *Xa25*, the characteristics of *xa25*-encoded protein and its expression pattern in the interaction between rice and *Xoo* indicate that the resistance mechanism mediated by *xa25* is different from most R proteins (Hutin et al., 2016).

### Xa27

*Xa27*, derived from *Oryzae* minuta, has broad-spectrum resistance to *Xoo*. *Xa27* was introduced into the susceptible variety “IR24” by hybridization and backcross, and the near isogenic line IRBB27 was constructed, and *Xa27* was isolated by map cloning strategy (Gu et al., 2004). *Xa27* and avrXa27 are the first combinations of resistance genes and corresponding nontoxic genes cloned from rice. *Xa27* has no intron and only one exon, which is a non-intragenic gene. Induced by the coding product of the non-toxic gene avrXa27 interacting with it, the gene encodes a protein consisting of 113 amino acids and containing a α-helix domain. The resistance and susceptibility alleles of *Xa27* also encode the same protein (Gu et al., 2005). The difference between the two genes is that the *xa27* promoter has a 10 bp sequence upstream of the promoter ATG, and 25 bases upstream of the TATA frame.

Although the resistance allele and susceptible allele of *Xa27* encoded the same protein, *Xa27* was only expressed in the adjacent cell tissues of rice infection under the infection of *X. oryzae* containing avrXa27. This indicated that the induced expression of *Xa27* was not induced by the system, but by the pathogen, and was only induced by the non-affinity strain containing avrXa27. This mechanism acts as a local defense and belongs to non-systematic defense. In addition, the *Xa27* infiltration system could resist the BB strain that originally infected IRBB27, and the promoter replacement experiment proved that the expression difference of *Xa27* in the resistant and susceptible parents was controlled by the promoter (Gu et al., 2005). *Xa27* gel block test showed that the affinity between *Xa27* promoter and AvrXa27 was at least 100 times that between *xa27* promoter and AvrXa27. When *Xa27* was driven by the PR1 gene promoter, the plants were resistant to both compatible and non-compatible pathogens, and the secondary cell wall of vascular bundle was thickened at the infection site, suggesting that *Xa27* might resist the invasion of pathogens by increasing the thickness of the secondary cell wall of vascular bundle. In order to verify the accuracy of the speculation, further subcellular localization found that XA27 may be transported to the vascular bundle cavity and the xylem parenchyma cell wall by cell exocytosis and accumulate more XA27 in vascular tissue. When the amino acid anchor signal sequence of XA27 was mutated, XA27 could not be subcellularly located and rice plants lost the ability to resist disease (Wu et al., 2008b).

### xa41

The *xa41* gene is a recessive resistance gene identified in African wild rice. It was found by analyzing the promoter polymorphism of *OsSWEET14*, the main susceptible gene of BB in 169 rice germplasm resources (Hutin et al., 2016). The 18 bp deletion in the promoter region of *OsSWEET14* gene represents a new resistance allele, named *xa41*. The recessive gene *xa41* and its allelic dominant gene *Xa41* also encode MtN3/saliva family proteins. The promoter of *Xa41* contains four binding sites of TALE (AvrXa7, Tal5, PthXo3, and TalC), which can be induced to express, so that the plant is susceptible (Yu et al., 2011). *xa41* is the 18 bp deletion allele of *Xa41* at the promoter, and the 18 bp deletion overlaps with the recognition sites of AvrXa7 and Tal5, thus producing resistance to *Xoo* strains containing these two TALEs (Streubel et al., 2013).

The SWEET susceptible gene families *Xa13*, *Xa25*, and *Xa41* were induced to express due to the presence of TALE binding elements in the promoter, which were identified by the pathogen. This part of the susceptible genes effectively obtained breeding materials with broad-spectrum disease resistance through gene editing technology (Oliva et al., 2019; Xu et al., 2019). In the future, such susceptible genes can be directly edited in high-yield and high-quality varieties or super hybrid rice parents to create new varieties with high yield and high resistance in large scale.

### Xa2/Xa31, Xa14, and Xa45

Previously, several major resistance genes such as *Xa2*, *Xa14*, and *Xa31* were located near the cloned *Xa1* gene, but the cloning of these genes has not been completed. Recently, *Xa2*, *Xa14*, *Xa31*, and *Xa45* were successfully cloned (Ji et al., 2020). These four genes, as alleles of *Xa1*, are all distributed on chromosome 4 and also encode NLR proteins, indicating the existence of gene duplication and functional evolution. *Xa2* and *Xa31* have the same sequence, which is actually the same gene. These genes are highly conserved and structurally similar at nucleotide and predicted amino acid levels. The main difference is that the repeat numbers of 93 amino acid residues at the C-terminal of protein are different. For example, the repeat numbers of XA14 and XA45 are 4 and 7, respectively.

*Xa1* gene is a member of NBS-LRR plant disease resistance genes (Yoshimura et al., 1998). The analysis of disease resistance genes near *Xa1* gene on the long arm of chromosome 4 reveals that the region is rich in NBS-LRR sequences (Freddy and Nishimura, 2018). *Xa1* and its alleles *Xa2*, *Xa14*, *Xa31*, and *Xa45* are a class of NLR genes, and the mediation of its disease resistance mechanism is also related to TALE. The NLR protein encoded by *Xa1* had specific resistance to BB. XA1 stimulated resistance to BB by identifying several TALEs including PthXo1, Tal4, and Tal9d. Further studies have found that truncated interference TALE (iTALE) lacking transcriptional activation domain can interfere with the resistance given by XA1 (Ji et al., 2016). The resistance spectrum of *Xa1* alleles was also inhibited to some extent by iTALEs, and the resistance spectrum of *Xa2*, *Xa31*, and *Xa14* to *Xoo* was different from that of *Xa1*, but also inhibited by iTALEs. This is the first report that the resistance genes inhibited by iTALE can mediate different resistance. These genes encode NBS-LRR proteins, and the key to determining the main difference is the functional domain of LRR at the carboxyl end (Biaoming et al., 2020).

## Research Prospect

### Breeding and Utilization of Cloned Rice BB Resistance Genes

Up to now, more than 40 rice BB resistance genes have been discovered, but most of the resistance genes are inevitably controlled by recessive genes, the control spectrum is narrow and the resistance is weak. Therefore, there are few resistance genes that can be put into practical production. In the actual breeding practice, the widely used BB resistance genes include *xa5*, *xa13*, and *Xa23*. In recent years, single-gene rice varieties have been planted in different degrees throughout the country. In the long run, the genetic structure of local rice pathogens will be changed, and mutant new races will be produced to infect the original resistance source, resulting in the disappearance of the resistance of the original varieties and bringing serious economic losses to the local crops. It has been reported that the application of single antigen will produce new *Xoo* strains in some areas. For example, Chinese race VIII that can cause *Xa21* disease was found in Guangdong, China (Zhang, 2005).

Therefore, it is not limited to the use of one resistance gene, but the comprehensive utilization of multiple resistance genes has become an effective means of breeding and production. Different rice BB resistance genes are polymerized into the same variety through reasonable comprehensive utilization, rotation, and mixed utilization, so that the target crops can obtain better resistance (Dai et al., 2010). The common forms of gene polymerization are two-gene polymerization, three-gene polymerization, and four-gene polymerization. The common forms of gene combination are double dominant gene combination and one recessive gene combination. The common gene binding forms in China are: *Xa4* + *Xa21*, *Xa21* + *Xa23*, *Xa7* + *Xa21*, *Xa4* + *Xa21* + *Xa23*, and *Xa4* + *xa5* + *xa13* + *Xa21*. For example, the *Xa4* + *xa5* polymerization system was developed by MAS technology (Cao et al., 2007). The *xa5* + *xa13* + *Xa21* polyline bred by molecular marker-assisted backcross breeding strategy showed high resistance (Pradhan et al., 2015). The resistance genes *Xa21* and Pi54 to BB and blast were polymerized into the restorer line RPHR-1005, and the progeny materials with high resistance to BB and blast were obtained by marker-assisted backcross breeding (Kumar et al., 2016). These genes showed high resistance and broad-spectrum characteristics by polymerization or combination.

In summary, the existing breeding practices have proved that the polymerization of different resistance genes into the same variety can enhance the resistance of varieties to varying degrees, broaden the resistance spectrum of varieties, and delay the decline of resistance of varieties through the interaction between resistance genes.

### Continuous Cloning of Novel Rice BB Resistance Genes

The resistance of rice and the pathogenicity of BB is a process of co-evolution and long-term competition. The change of any party will cause the change of the other party. The mutation of the physiological race of BB will cause the variation of rice, and the external pressure on rice will also cause the mutation of the physiological race of BB. In order to effectively control the mutation and infection of rice BB, it is necessary to continuously explore new resistant resources (Lee et al., 2011).

*Xa1* gene was isolated and cloned by Japanese researchers in 1998 (Yoshimura et al., 1998). It is reported that many full-length TALEs can trigger *Xa1*-mediated resistance. However, interfering iTALE in pathogen PXO99A interferes with *Xa1*-mediated resistance. iTALE exists in many *Xoo* strains, resulting in the narrowing of the resistance of *Xa1*, so the application of *Xa1* is not much (Ji et al., 2016). The genes such as *Xa4* and *xa5* excavated later are more resistant and broad-spectrum than *Xa1* and have been widely used in practical production (Li et al., 1999). The main rice varieties in China have also introduced resistance genes such as *Xa3*, *Xa21*, and *Xa23*, which once effectively controlled BB and made the incidence of BB lighter and lighter. However, with the gradual warming of the global environment and the continuous variation of BB, new pathogenic varieties have emerged, leading to the gradual loss of resistance of main rice varieties. However, under the environmental conditions of high-temperature stress, the disease resistance pathway mediated by the disease resistance gene *Xa7* can more effectively limit the growth and reproduction of BB compared with genes such as *Xa3*, *Xa4*, Xa5, and *Xa10* (Cohen et al., 2017). *Xa7* has been successfully cloned recently, and under the condition of global warming, *Xa7* reflects the stronger breeding value of its own genes (Chen et al., 2021).

It can be seen that although significant progress has been made in molecular breeding for BB resistance in China, the variation of pathogen itself and the stress of adverse environmental conditions still threaten the effectiveness of existing resistance genes. The disease resistance of rice is essentially the result of the interaction between the target crop rice and pathogenic bacteria under specific conditions and the two evolve and compete with each other. In the long-term interaction process, due to the continuous emergence of new pathogenic strains and the evolution of pathogenic physiological races, the effectiveness of existing rice resistance genes continues to decrease. Therefore, continuous cloning of new genes with broad-spectrum and long-lasting disease resistance from rice to resist the infection of rice BB is the basis and key to the success of disease resistance breeding.

### Mining of Resistance Genes to BB in Wild Rice

Due to the unique climatic conditions and geographical location, wild rice and local rice resources in China are extremely rich, and many excellent genetic traits have been retained in the long-term self-evolution for many years. Particularly, wild rice resources not only show the characteristics of high resistance to BB, but also are partially immune.

At present, there are many effective genes for resistance to BB cloned from wild rice resources. For example, *Xa21* is the first cloned gene for resistance to BB in rice, which is from wild rice with long stamens (Park et al., 2012). *Xa23* is a broad-spectrum resistance gene to BB found in long-acting wild rice (Zhou et al., 2015). *Xa27* is derived from tetraploid small-grain wild rice (Bimolata et al., 2013). *xa41* is identified on wild rice in Africa. These resistance genes excavated from wild rice germplasm resources have broad-spectrum resistance to *Xoo*. For example, *Xa21*, *Xa23*, *Xa27*, and other resistance genes excavated from wild rice varieties have been widely used in production practice and achieved ideal resistance effect. In addition, there are also some resistance genes from wild rice: *xa32* in the new germplasm Y76 obtained by somatic hybridization between wild rice verrucous and cultivated rice from Xishuangbanna, Yunnan (Ruan et al., 2008); *Xa36* from Australian wild rice and cultivated rice hybrids (Miao et al., 2010); and *Xa38* from annual common wild rice (Ellur et al., 2016), etc. These genes have been mapped but not cloned and have broad application prospects.

The wild rice resources that have been explored and utilized and other resistance genes that have not been found are valuable breeding resources for rice resistance to BB. By revealing the resistance mechanism of wild rice and local rice, it is of great significance to enrich the resistance gene pool of rice and further clarify the genetic basis of disease resistance in rice cultivation.

### Expanding Gene Resistance Spectrum by Gene Editing

With the continuous warming of global climate, the continuous growth of population and the continuous reduction of cultivated land, the traditional agricultural production methods, and methods cannot meet the needs of the development of modern society. Therefore, there is an urgent need for scientific and technological innovation to improve crop varieties and traits. Through gene editing, specific target genes can be targeted modified, which can greatly make up for the deficiency of natural mutation and achieve the specific purpose required for production (Hassan et al., 2021).

The development of gene editing technology has experienced a long process, from the initial zinc finger nuclei (ZFNs), transcription activator-like effector nucleases (TALENs) to CRISPR/cas9. The editing efficiency and operability of gene editing technology have been continuously improved, which has attracted extensive attention. It is worth noting that by knocking out the promoter and other key elements of host susceptibility genes, the expression of crop susceptibility genes by pathogenic microorganisms can be regulated and disease-resistant germplasm can be created. For example, knocking out the EBE region of the promoters of susceptibility genes OSSWEET11, OSSWEET 14, and OSSWEET 13 in rice leads to the inability of TaL effector protein to activate the expression of relevant susceptibility genes, resulting in extensive resistance of rice to BB (Xu et al., 2019).

The breakthrough of gene editing technology represented by CRISPR/cas9 makes the current gene operation more mobile and feasible. CRISPR/cas9 gene editing technology is simpler and cheaper than traditional breeding methods (Wang et al., 2014a). The research shows that CRISPR/cas9 gene editing technology has greatly improved the gene editing efficiency of tomato, wheat, rice, soybean, and other crops. The second generation CRISPR/cas9 gene editing technology gradually formed in the later stage is easier to realize single-base editing. Single-base editing can realize base substitution from cytosine (c) to thymine (T) and adenine (a) to guanine (g; Komor et al., 2016). For example, by fusing RNA adenylate deaminase with cas9 notch enzyme, the conversion of target base a to G can be realized (Gaudelli et al., 2018). The translation of genes can be terminated in advance by converting the codon encoded by amino acids into a stop codon (Kuscu et al., 2017). These new single-base editing strategies greatly expand the application scope of gene editing technology and lay a foundation for obtaining new disease resistant materials.

Although the low editing efficiency and high miss rate still exist in gene editing (Qin et al., 2020), which limits the large-scale application of this technology, the rapid development of gene editing technology in the past two decades can now create genetic variations with high accuracy and specificity. Therefore, gene editing technology is also widely used in polyploid breeding, germplasm resource innovation, and molecular design breeding. It is believed that with the maturity of this technology, it will play a greater role in crop breeding.

## Author Contributions

YY, YuhZ, JS, WL, and JC contributed to conceptualization. YY, YuhZ, JS, WL, XC, XW, JZho, CYu, JW, SW, XY, YujZ, JZhu, and CYa were involved in collecting and reading the literature. YY, YuhZ, JS, WL, XC, XW, JZho, CYu, JW, SW, and BZ contributed to writing—original draft preparation. YY, YuhZ, JS, WL, and JC were involved in writing—review and editing. All authors contributed to the article and approved the submitted version.

## Funding

This research was funded by Zhejiang Provincial Key Research and Development Plan (2019C02006), the National Natural Science Foundation of China for young scholar (31101208), the Key Program of Zhejiang Provincial Foundation for Natural Science (LZ16C130002), Zhejiang Fundamental Public Welfare Research Program (LGN19C140008), and State Key Laboratory for Managing Biotic and Chemical Threats to the Quality and Safety of Agro-products (2010DS700124-ZZ1907).

## Conflict of Interest

The authors declare that the research was conducted in the absence of any commercial or financial relationships that could be construed as a potential conflict of interest.

## Publisher’s Note

All claims expressed in this article are solely those of the authors and do not necessarily represent those of their affiliated organizations, or those of the publisher, the editors and the reviewers. Any product that may be evaluated in this article, or claim that may be made by its manufacturer, is not guaranteed or endorsed by the publisher.
